# Multiple hepatic inflammatory pseudotumors with elevated alpha-fetoprotein and alpha-fetoprotein lectin 3 fraction with various PET accumulations: a case report

**DOI:** 10.1186/s40792-021-01188-6

**Published:** 2021-04-28

**Authors:** Masataka Maruno, Katsunori Imai, Yosuke Nakao, Yuki Kitano, Takayoshi Kaida, Kosuke Mima, Hiromitsu Hayashi, Yo-Ichi Yamashita, Yoshiki Mikami, Hideo Baba

**Affiliations:** 1 Department of Gastroenterological Surgery, Kumamoto University Graduate School of Medical Sciences, Kumamoto University, 1-1-1 Honjo, Chuo-ku, Kumamoto, 860-8556 Japan; 2 Department of Diagnostic Pathology, Kumamoto University Hospital, Kumamoto, Japan

**Keywords:** Hepatic inflammatory pseudotumor, Alpha-fetoprotein, Tumor markers, Hepatocellular carcinoma

## Abstract

**Background:**

Hepatic inflammatory pseudotumor (IPT) is a rare, benign, tumor-like lesion. Because there are no characteristic laboratory markers or radiological features, hepatic IPT is often misdiagnosed as a malignant neoplasm such as hepatocellular carcinoma (HCC).

**Case presentation:**

A 68-year-old man with liver dysfunction due to chronic hepatitis C virus infection and alcoholic liver disease presented with hepatic tumors in segments III and VIII. The levels of serum alpha-fetoprotein (AFP) and its *Lens culinaris* agglutinin-reactive fraction, AFP lectin 3 (AFP-L3), were elevated to 822.8 ng/ml and 75.2%, respectively. The tumor showed contrast enhancement on contrast-enhanced computed tomography and various accumulation on positron emission tomography. Based on these biological and imaging features, HCC was suspected, and we performed laparoscopic partial hepatectomy for these two tumors. Pathological diagnosis revealed that both tumors were hepatic IPTs with no malignant characteristics. After hepatectomy, the serum AFP and AFP-L3 levels decreased to the normal range.

**Conclusion:**

We report a very rare case of hepatic IPT with elevated serum AFP and AFP-L3, mimicking HCC. Clinicians should include this rare neoplasm in the differential diagnoses of hepatic tumors even when the serum markers for HCC are elevated.

## Background

Hepatic inflammatory pseudotumor (IPT) is a rare benign tumor-like lesion that was first described in 1953 by Pack and Baker [1]. There are many theories regarding the etiology of this disease, including bacterial and viral infections, autoimmune diseases, and cholangitis, but this is not yet well understood [2, 3]. There are no specific laboratory markers or radiographic features for hepatic IPT [3], as a result, hepatic IPT has often been misdiagnosed as a malignant tumor, and surgical resection has been performed. Herein, we report a case of hepatic IPT misdiagnosed as hepatocellular carcinoma (HCC) due to the elevated serum levels of alpha-fetoprotein (AFP) and its *Lens culinaris* agglutinin-reactive fraction AFP lectin 3 (AFP-L3), and radiological findings.

## Case presentation

**Fig. 1:**
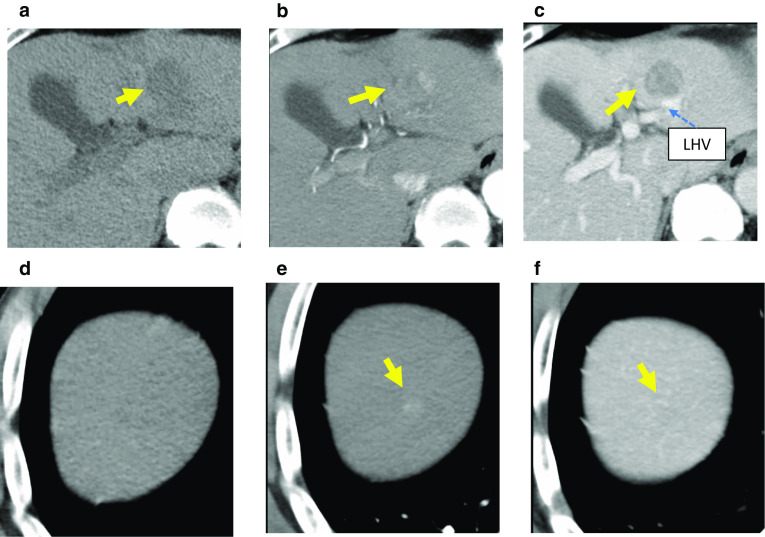
Contrast-enhanced computed tomography (CT) revealed the presence of lesions in the patient’s liver. **a** A low-density mass, 24 mm in size, in segment III on plain CT. **b** Enhancement of the lesion in the early phase was noted. **c** In the late phase, washout was observed. The tumor was in contact with the left hepatic vein (LHV). **d** The tumor in segment VIII was not clear on plain CT. **e** An 8-mm tumor was also present in segment VIII of the liver showing contrast enhancement. **f** Washout was noted in the delayed phase

**Fig. 2:**
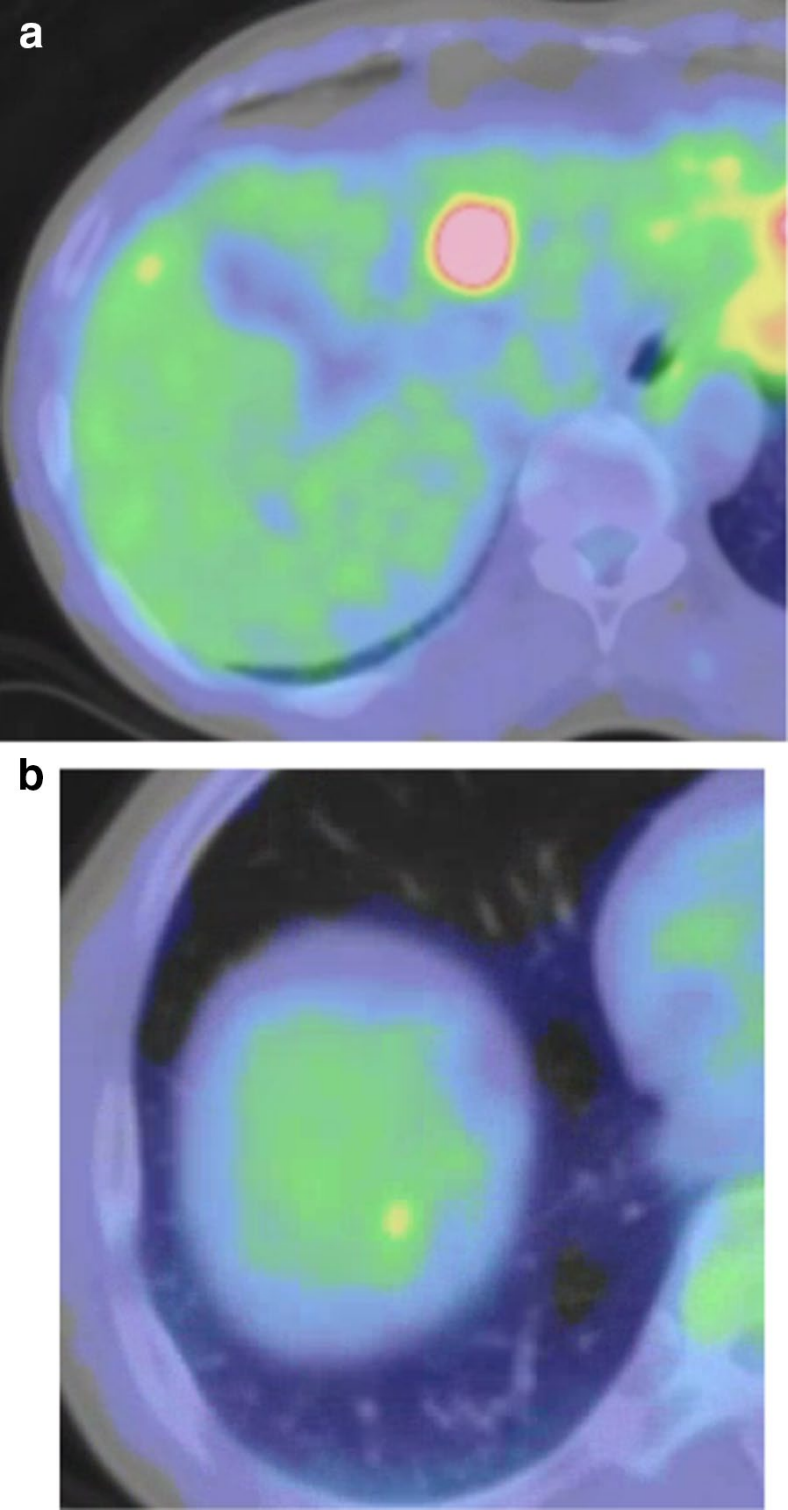
Fluorine-18-fluorodeoxyglucose positron emission tomography (FDG-PET) findings. **a** The segment III lesion appeared abnormal on FDG-PET. **b** The segment VIII lesion did not show an abnormal uptake

A 68-year-old man, who had been undergoing medical treatment for liver dysfunction due to chronic hepatitis C virus (HCV) infection and alcoholic liver disease, was found to have a liver tumor measuring 25 mm in segment III on abdominal ultrasonography and was referred to our hospital for further evaluation. He was on antihypertensive medication and had undergone open reduction and internal fixation with metal plates for the fracture of his left upper extremity. He was a past smoker, with a daily alcohol intake of 80 g for 42 years. Laboratory analysis revealed the following results: aspartate aminotransferase, 46 U/L (normal range: 10–40 U/L); alanine aminotransferase, 51 U/L (normal range: 5–45 U/L); alkaline phosphatase, 210 U/L (normal range: 100–325 U/L); and gamma-guanosine triphosphate, 27 U/L (normal range < 30 U/L). Serum AFP and AFP-L3 were 822.8 ng/mL (normal range < 10 ng/mL) and 75.2% (normal range < 35%), respectively. Levels of protein induced by vitamin K absence or antagonist II, carcinoembryonic antigen, and carbohydrate antigen 19-9 were within normal limits. Serological tests for hepatitis viruses showed that hepatitis B surface antigen was negative, but hepatitis B surface antibody, hepatitis B core antibody, and HCV antibody were positive. There was no anemia or elevation of white blood cell count and C-reactive protein level. Coagulation profile, total protein, albumin, and bilirubin levels (both total and direct) were normal. Contrast-enhanced computed tomography (CT) showed a 24-mm mass in segment III of the liver adjacent to the left hepatic vein, and an 8 mm mass in segment VIII. These lesions showed weak enhancement in the early phase and demonstrated washout of the contrast in the delayed phase (Fig. 1). The lesion in segment III appeared abnormal on fluorine-18-fluorodeoxyglucose positron emission tomography (FDG-PET) with a high standardized uptake value of 10.9, however, the segment VIII lesion did not show this uptake pattern (Fig. 2). Magnetic resonance imaging could not be performed because of the presence of the metal plates in his arm. Based on the findings of HCV infection, elevated serum AFP and AFP-L3, and radiological imaging of the liver, HCC was suspected. Thus, laparoscopic left lateral sectionectomy and partial hepatectomy for segment VIII were performed.

**Fig. 3:**
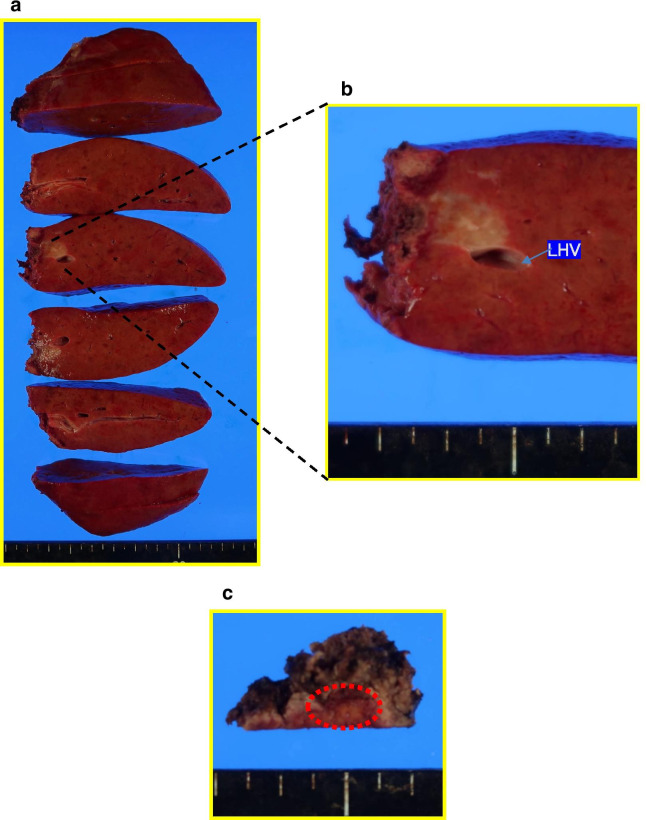
Gross appearance of the lesions removed via laparoscopic hepatectomy. **a** White nodules in segment III. **b** As indicated by the preoperative imaging studies, the tumor was in contact with the left hepatic vein. **c** A white nodule was also found in segment VIII

**Fig. 4:**
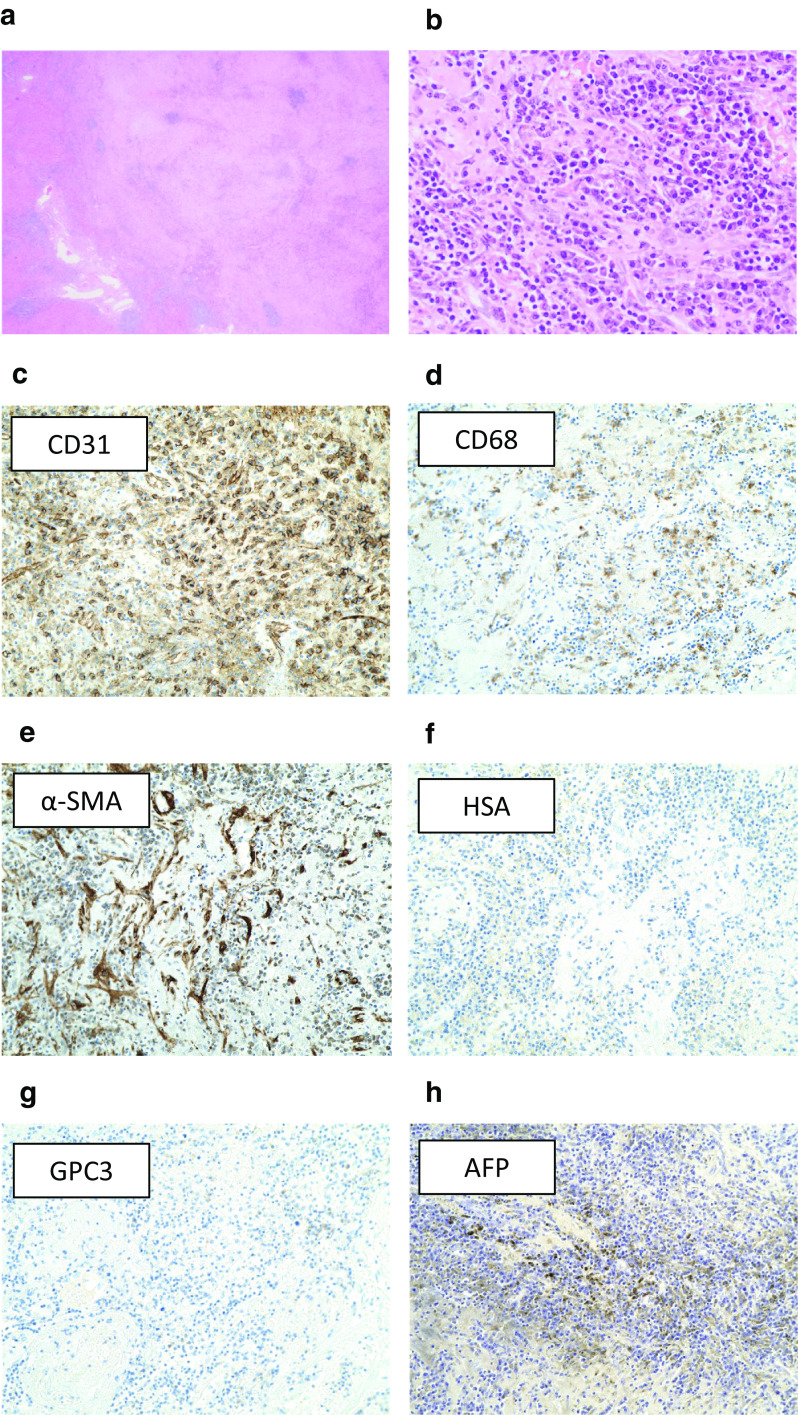
Histopathological findings. **a** On low-powered magnification, lymphatic follicle formation and glass-like fibrosis could be seen. **b** On high-powered magnification, infiltration of plasma cells and lymphocytes could be observed. Malignant features were not present. **c–e** Immunohistochemistry for CD31 (**c**), CD68 (**d**), alpha-smooth muscle actin (α-SMA) (**e**), human serum albumin (HSA) (**f**), glypican 3 (GPC3) (**g**), and alpha-fetoprotein (AFP) (**h**). Tumor cells were diffusely positive for CD31, and partially positive for CD68 and α-SMA, whereas human serum albumin and glypican 3 were negative. There were some tumor cells positive for AFP

Macroscopically, the resected specimen showed hard white nodules, not like HCC, in approximately the same location that was indicated by the imaging studies (Fig. 3). Histopathological analysis showed that both tumors consisted of fibrous tissue infiltrated by inflammatory cells, mainly plasma cells (Fig. 4a, b). In immunohistopathological analysis, tumor cells were diffusely positive for CD31, and partially positive for CD68 and alpha-smooth muscle actin, whereas human serum albumin and glypican 3 were negative (Fig. 4c–g), suggesting that the tumor cells contain lymphocytes and macrophage but not cells derived from hepatocyte. And, there were some tumor cells positive for AFP in immunohistochemical staining (Fig. 4h). Non-tumorous liver tissues were noted to be A2F3–4 based on New Inuyama Classification [4]. Malignant cells, including HCC cells, were not detected. The lesions were finally diagnosed as hepatic IPT.

The patient had no postoperative complications and was discharged 7 days after surgery. AFP and AFP-L3 levels decreased to 13 ng/mL and 38.9%, respectively, 1 week after of surgery, and returned to normal levels 3 months later, to 5.5 ng/mL and < 0.5%, respectively. These tumor markers were not elevated during the 1-year follow-up.

## Discussion

In the present case, the preoperative diagnosis was HCC because of the patient’s background chronic liver disease, elevated serum AFP and AFP-L3, and radiological findings such as high–low pattern. In hepatic IPTs, although inflammatory markers, including C-reactive protein and leukocyte count, and liver enzymes are sometimes elevated, tumor markers are usually normal [5]. AFP is a well-known tumor marker for HCC [6]. However, AFP levels also increase in non-malignant hepatic diseases such as acute/chronic hepatitis and cirrhosis, or in normal pregnancy [6, 7]. AFP-L3, an isoform of AFP, has a greater sensitivity and specificity for detecting HCC [6]. A similar case of hepatic IPT with elevated AFP and AFP-L3 levels (102 ng/mL and 85.4%, respectively) was reported [8]. However, the AFP level in the present case was approximately eight times higher than that reported in the abovementioned case. In the present case, immunohistochemical analysis showed that the tumor cells were positive for AFP, and the serum AFP level decreased dramatically to the normal level after resection of the tumor. These facts suggest that the tumor cells apparently produced AFP.

Radiological findings of IPT are also non-specific. CT scan often shows a low-density mass [3, 9–11]. On contrast-enhanced CT, IPT shows a variable pattern of enhancement. Generally, it shows peripheral enhancement in the delayed phase, similar to metastatic liver tumors [3, 5]. However, some studies have reported lesions with early phase enhancement and washout in the delayed phase, similar to HCC [8, 12]. The lesions in the present case also showed this pattern of enhancement. FDG-PET is not commonly performed; however, similar to the present case, there is a previous report of an IPT with abnormal metabolic activity on FDG-PET [12]. HCC is also known to have different imaging findings on FDG-PET depending on the degree of differentiation [13, 14]. In addition, hepatic IPT is usually detected as a solitary tumor [3, 5, 8, 11, 12]. In contrast, our patient had multiple IPTs in the liver, and it was reported that some patients with multiple lesions had both IPTs and malignant tumors [9]. Based on these imaging findings, we suspected intrahepatic metastasis of poorly differentiated hepatocellular carcinoma.

Once a definitive diagnosis is obtained, conservative management is the treatment of choice for IPT. However, as mentioned above, differentiating IPT from malignancy, on the basis of noninvasive diagnostic methods, is very difficult. In fact, surgical resection is frequently selected as the treatment of choice for hepatic IPTs because these tumors were suspected to be malignant tumors, preoperatively, in many cases [5, 8, 11, 15]. However, spontaneous regression has been reported in a few cases, and if this occurs, the indication for surgery should be carefully reconsidered [10]. If the patient has chronic hepatitis or cirrhosis and HCC-suspected lesion with elevated tumor markers such as AFP and AFP-L3, like the present case, the patient would be recommended surgical resection. However, we should keep in mind the possibility of benign tumors such as IPT. Although preoperative diagnosis of IPT is very difficult at this point, a careful clinical examination with the recognition of the possibility of IPT may help prevent unnecessary or at least, excessive surgical intervention.

## Conclusions

In conclusion, we encountered a rare case of multiple hepatic IPTs with elevated AFP and AFP-L3 levels, mimicking HCC. We should keep in mind that even if these tumor markers, especially AFP-L3, are elevated, it does not always indicate the presence of HCC, as in our reported case. Our report also shows that hepatic IPT can be one of the benign tumors associated with elevated AFP and AFP-L3 levels.

## Data Availability

All data generated or analyzed during this study are included in this published article.

## Abbreviations

AFP: Alpha-fetoprotein
AFP-L3: Alpha-fetoprotein lectin 3
CT: Compute tomography
HCC: Hepatocellular carcinoma
HCV: Hepatitis C virus
FDG-PET: Fluorine-18-fluorodeoxyglucose positron emission tomography
IPT: Inflammatory pseudotumor
